# Local Ca^2+^ Signaling Domains: The Balancing Act between CRAC Channels and Plasma Membrane Ca^2+^ Pumps

**DOI:** 10.1093/function/zqac045

**Published:** 2022-09-05

**Authors:** Ole H Petersen

**Affiliations:** School of Biosciences, Cardiff University, Museum Avenue, Cardiff CF10 3AX, UK

**Keywords:** calcium signalling, local calcium domains, orai1 calcium channels, calcium release activated calcium channels, calcium pumps, control of gene expression

## A Perspective on “Plasma Membrane Ca^2+^ ATPase Activity Enables Sustained Store-operated Ca^2+^ Entry in the Absence of a Bulk Cytosolic Ca^2+^ Rise”

We have known for a long time that the physiologically important cytosolic Ca^2+^ signals, which control or influence an astonishing range of cellular processes, are localized in specific subcellular areas. Astonishingly subtle temporal and spatial regulations of Ca^2+^ transport proteins have been shown to elicit precise sequences of processes that have real physiological importance.^1^^,^^2^ One can distinguish between micro- and nanogradients of Ca^2+^. Microgradients occur within spatial dimensions of 1–10 µm, and it became possible to visualize such events many years ago. Nanogradients, as the name implies, occur within much smaller spatial dimensions, typically decaying with space constants of tens of nanometers. Because the nanogradients may also be short-lasting, it is particularly challenging to visualize them.^1^ In general, physiological Ca^2+^ signals are oscillatory and a locally elevated [Ca^2+^] returns quickly to the basal level due to diffusion away from the domain, reuptake by Ca^2+^ pumps into the endoplasmic reticulum (ER), uptake via the Ca^2+^ uniporter into the mitochondria, and extrusion to the extracellular space via Ca^2+^ pumps in the plasma membrane.^1^^,^^2^

In the electrically nonexcitable cells, the original focus was predominantly on local cytosolic Ca^2+^ signal generation near IP_3_ receptors (IP_3_Rs) on the ER membrane.^3^^,^^4^ One of the consequences of eliciting Ca^2+^ release from the ER via IP_3_Rs is opening of Ca^2+^-release activated Ca^2+^ (CRAC) channels in the plasma membrane.^5^ Tullio Pozzan and his collaborators provided early evidence for local domains of elevated [Ca^2+^] beneath the plasma membrane, particularly during activated Ca^2+^ influx.^6^ Further studies of the Ca^2+^ domains in the subplasmalemmal space near CRAC channels and the processes initiated by such local signals have provided important insights into the control of many different physiological events.^1^^,^^5^

As mentioned above, it has generally been thought that physiological Ca^2+^ signals are mostly oscillatory, but, in an important article published in this issue of *Function*,^7^ Parekh and collaborators have now provided compelling evidence for the view that CRAC channels can remain open and create a sustained elevated [Ca^2+^] in the local cytosolic space near the inner mouth of the CRAC channels, in spite of the bulk cytosolic Ca^2+^ concentration only being minimally elevated due to the operation of the plasma membrane Ca^2+^ ATPase.

The experimental arrangement used by Barak *et al*.^7^ is, inevitably, nonphysiological, but has the considerable advantage of isolating a few key components of the Ca^2+^ signaling system, thereby creating a situation that allows rigorous testing of the hypothesis that a sustained elevated subplasmalemmal [Ca^2+^] can be maintained with only minimal elevation of the bulk cytosolic [Ca^2+^], thus creating a standing Ca^2+^ gradient. In HEK293 cells, thapsigargin was used to arrest the ER Ca^2+^ pumps. Ca^2+^ therefore leaked out of the ER, gradually emptying the store and activated CRAC channels of the Orai1 type in the plasma membrane. In this situation, with the ER Ca^2+^ pumps out of action, the only transport proteins capable of removing excess Ca^2+^ from the cytosol would be the Ca^2+^ pumps in the plasma membrane. In addition to standard measurements of the bulk cytosolic [Ca^2+^], Barak *et al*.^7^ were able to assess the local [Ca^2+^] in the subplasmalemmal space near the CRAC (Orai1) channels by expressing an Orai1 construct in which the Ca^2+^-sensitive fluorescent protein GECO was tagged to the N-terminus of the channel. The key experiments showed that thapsigargin elicited major rises in [Ca^2+^] both in the cytosol at large as well as close to the Orai1 channels but, whereas the bulk cytosolic [Ca^2+^] returned to a near prestimulation basal level within about 10–15 min, the local [Ca^2+^] close to the Orai1 channels remained markedly elevated even 25 min after the start of thapsigargin application. In spite of the relatively fast return of the bulk cytosolic [Ca^2+^] to the basal level, the elevated subplasmalemmal [Ca^2+^] was shown to be capable of driving the slowly developing migration of NFAT into the nucleus.^7^ Inhibition of the plasma membrane Ca^2+^ pumps, either by external La3^+^ or by increasing the external pH to 11, very effectively prevented the rapid return of the bulk cytosolic [Ca^2+^] to the basal level. In contrast, marked reduction of the external [Na^+^] had hardly any effect, indicating that the Na^+^–Ca^2+^ exchanger in the plasma membrane does not play any important role.^7^ Clearly, in an experimental situation with nonfunctional Ca^2+^ pumps in the ER, the plasma membrane Ca^2+^ pumps are by far the most important elements disposing of excess cytosolic Ca^2+^, in complete agreement with previous studies on electrically nonexcitable cells.^1^^,^^2^

The apparent prolonged existence of nanodomains with a high [Ca^2+^] in the subplasmalemmal space^7^ would be difficult to explain if the Ca^2+^ pumps in the plasma membrane were colocalized with the CRAC channels. Indeed, Barak *et al*. show that during thapsigargin-elicited Ca^2+^ inflow, there is little colocalization between these two types of Ca^2+^ transporters in the HEK293 cells.^7^ In polarized epithelial cells, for example, the pancreatic acinar cells, which have a clear physiological role in the body, there can be an almost complete separation of these transporters, as the plasma membrane Ca^2+^ pumps are concentrated in the apical membrane, whereas the CRAC channels reside in the baso-lateral membrane, an arrangement of great physiological importance.^2^ It has also become clear that activated CRAC channels are not uniformly distributed throughout the baso-lateral membrane, but clustered in certain areas.^2^ This was also shown to be the case in the HEK293 cells.^7^

In order to explain the sustained difference between [Ca^2+^] in the subplasmalemmal domains and the bulk of the cytosol, it is necessary to assume a major “resistance” to Ca^2+^ movements. It has been known for a long time that Ca^2+^ diffusion in the cytosol is severely restricted.^1^ In the pancreatic acinar cells, for example, Ca^2+^ diffuses more readily inside the ER than in the cytosol, as the Ca^2+^ binding capacity in the cytosol is orders of magnitude higher than in the lumen of the ER.^8^

Are the conclusions from the experiments of Barak *et al*.,^7^ with nonfunctional ER Ca^2+^ pumps in HEK293 cells, relevant to physiology? Clearly, the ER Ca^2+^ pumps, which overall are more powerful than the plasma membrane Ca^2+^ pumps,^2^ can have a strong influence on [Ca^2+^] in both local domains and the bulk of the cytosol, but, as pointed out by Rizzuto and Pozzan,^1^ the long-term steady-state [Ca^2+^] in the cytosol depends exclusively on the equilibrium between Ca^2+^ entry and Ca^2+^ outflow across the plasma membrane. By exploiting a conceptually simple experimental situation, Barak *et al*.^7^ have been able to demonstrate how Ca^2+^ pumps in the plasma membrane can protect the bulk of the cytosol against toxic elevations of [Ca^2+^] while still allowing a sustained local [Ca^2+^] elevation near the plasma membrane to drive gene expression. This is an achievement of real physiological importance.

## Data availability

There are no data presented in this editorial/perspective.
